# Touch Restriction Versus Free‐Play Small‐Sided Games in Soccer: Acute Effects on Serum BDNF, Executive Function, and Technical Actions

**DOI:** 10.1002/ejsc.70242

**Published:** 2026-08-22

**Authors:** Yakup Zühtü Birinci, Serkan Pancar, Yusuf Soylu, Emre Sarandöl, Hasan Şimşek, Şenay Şahin, Michael C. Rumpf

**Affiliations:** ^1^ Faculty of Sport Sciences Bursa Uludağ University Bursa Türkiye; ^2^ Faculty of Sport Sciences Aksaray University Aksaray Türkiye; ^3^ Faculty of Sport Sciences Tokat Gaziosmanpasa University Tokat Türkiye; ^4^ Faculty of Medicine Bursa Uludağ University Bursa Türkiye; ^5^ Faculty of Medicine Aksaray University Aksaray Türkiye; ^6^ Department of Sport Science and Sport Friedrich‐Alexander University Erlangen‐Nürnberg Erlangen Germany; ^7^ Footballscience.net Dreieich Germany; ^8^ Sport Performance Research Institute New Zealand AUT‐University Auckland New Zealand

**Keywords:** brain, cognitive performance, football, game‐based training, neurotrophic factors

## Abstract

This study aimed to compare the acute effects of two 4v4 small‐sided game (SSG) formats, touch‐restricted SSG (SSGt) and free‐play SSG (SSGf), on serum brain‐derived neurotrophic factor (BDNF) levels, cognitive performance, and technical actions in young male soccer players. Thirty‐two young male soccer players completed two 4v4 SSG conditions in a randomized crossover design: SSGt (a maximum of two ball touches per possession) and an SSGf. Serum BDNF was measured before and after each session. Executive function was assessed with the Stroop task, cognitive load was evaluated with the Rating Scale of Mental Effort (RSME), and technical actions were analyzed through match performance metrics. Internal load was monitored using heart rate, rating of perceived exertion, and blood lactate levels. Serum BDNF increased significantly only in the SSGt condition, with higher post‐exercise values than in the SSGf condition. Mental effort was also significantly greater in SSGt. Executive function performance improved only in SSGt, as reflected by faster completion time in the incongruent color‐word interference condition, whereas errors and corrections did not differ between conditions. Moreover, SSGt showed higher numbers of successful passes and shots, fewer unsuccessful passes, and lost balls, whereas interceptions were higher in SSGf. In conclusion, 4v4 SSG with a touch restriction may be a useful strategy for shaping acute neurotrophic, executive‐function, and technical‐action responses in soccer players. These findings suggest that touch restrictions may help coaches broaden the training stimulus of SSG by increasing cognitive demands without reducing technical effectiveness.

**Trial Registration:**

This study was retrospectively registered at ClinicalTrials.gov (Identifier: NCT07395765)

## Highlights

- A maximum two‐touch rule during 4v4 small‐sided games elicited greater acute serum BDNF responses than free‐play small‐sided games in young male soccer players.
- Touch‐restricted small‐sided games increased perceived mental effort and improved Stroop performance, particularly under conditions of high interference.
- Touch restriction increased successful passing and shooting actions without reducing technical effectiveness, suggesting a practical way to enrich the cognitive stimulus of soccer‐specific training.

## 1 Introduction

Small‐sided games (SSG) are game‐based training formats that simulate the physical, technical, and tactical demands of competitive soccer (Sarmento et al. 2018) by manipulating so called task constraints (Davids et al. 2013) possibly consisting of one or a combination of the following: player numbers (F. M. Clemente et al. 2025; Rumpf et al. 2026), pitch size (Rumpf, Jäger, Clemente, et al. 2025), and game rules, such as touch restrictions, that is, 2‐touch limitation per player possession (Coutinho et al. 2022; Rumpf, Jäger, Altmann, et al. 2025). Touch restrictions alter the physical and technical involvement of players with no clear indication on tactical variables (Giménez et al. 2018; Rumpf, Jäger, Altmann, et al. 2025). Nevertheless, effects were observed regarding decision making, player interactions with team mates (Coutinho et al. 2022), and ball circulation (Sarmento et al. 2018). Touch‐restriction constrains the range of immediate action options available to the player in possession (Jäger et al. 2026) and players may need to select subsequent actions earlier, often before or immediately upon receiving the ball (Coutinho et al. 2022; Vilar et al. 2013). Under these conditions, players must continuously monitor teammates, opponents, and ball movement increasing the perceptual‐cognitive demands such as attentional control, visual scanning, and spatial awareness (Birinci et al. 2025; González‐Fernández et al. 2026) requiring to process relevant game information and commit to decisions within limited ball‐contact opportunities (Jäger et al. 2026; Rosten et al. 2025). However, the mechanisms underlying these cognitive responses remain unclear.

Exercise‐related cognitive benefits are thought to involve multiple biological pathways, including molecular and cellular processes linked to neuroplasticity (Herold et al. 2018; Stillman et al. 2016). At the molecular level, acute exercise may upregulate neurotrophic factors (Piepmeier and Etnier 2015) and myokines (Kim et al. 2019), thereby promoting neuroplasticity and supporting cognitive functions (Basso and Suzuki 2016). Among the potential neurobiological candidates, brain‐derived neurotrophic factor (BDNF) is particularly relevant as an exercise‐responsive neurotrophin implicated in neurocognitive adaptation (Knaepen et al. 2010). BDNF supports neuronal survival and synaptic plasticity by promoting dendritic growth, synaptogenesis, and long‐term potentiation (Park and Poo 2013), thereby contributing to learning‐ and memory‐related processes across the lifespan (Erickson et al. 2011). Acute responses to exercise are generally associated with increases in circulating BDNF, although the magnitude of this response appears to vary according to exercise intensity (Birinci et al. 2026; Szuhany et al. 2015), duration (Dinoff et al. 2017), modality (Behrendt et al. 2021), and task demands (Hung et al. 2018). This task‐demand perspective is also important in soccer, where successful performance depends not only on physical and technical abilities but also on executive functions that support information processing, decision‐making, attentional control, response inhibition, and cognitive flexibility under rapidly changing game conditions (Vestberg et al. 2012).

In soccer contexts, BDNF has also been discussed as a candidate biomarker reflecting training‐related stress and potentially sport‐relevant cognitive functioning (Andrzejewski et al. 2022; Gutiérrez ‐Vargas et al. 2021). However, evidence specific to SSG remains limited, with available studies reporting no significant acute changes in BDNF after 4v4 free‐play SSG (Birinci et al. 2025) or after a soccer‐specific session combining technical drills with 5v5 SSG (Williams et al. 2020). Although the latter was not specified in greater details, the exercise protocol of Birinci et al. (2025) in the initial 4v4 free play consisted of four 4‐min bouts without any task‐constraints. Because broader evidence suggests that combining physical and cognitive stimulation may provide a stronger stimulus for cognitive adaptation than either component alone (Herold et al. 2018; Mao et al. 2024), touch restriction may represent an ecologically valid soccer‐specific strategy for embedding cognitive stimulation within physical training. Considering the well‐established involvement of BDNF in exercise‐related neuroplasticity and executive‐function processes, determining whether a rule‐induced cognitive constraint within SSG is accompanied by acute serum BDNF responses and executive‐function changes would address an important gap in understanding the neurochemical context of soccer‐specific cognitive demands. Therefore, this study aimed to clarify whether imposing a touch restriction during 4v4 SSG influences (i) serum BDNF responses, (ii) executive‐function performance, and (iii) technical‐action performance compared with free play. We hypothesized that, compared with free play, the touch‐restricted format would elicit greater perceived mental effort, a larger acute serum BDNF response, and greater improvement in executive‐function performance. Technical actions were additionally examined to determine whether the two formats produced distinct game‐behavior profiles.

## 2 Methods

### 2.1 Experimental Approach

**FIGURE 1:**
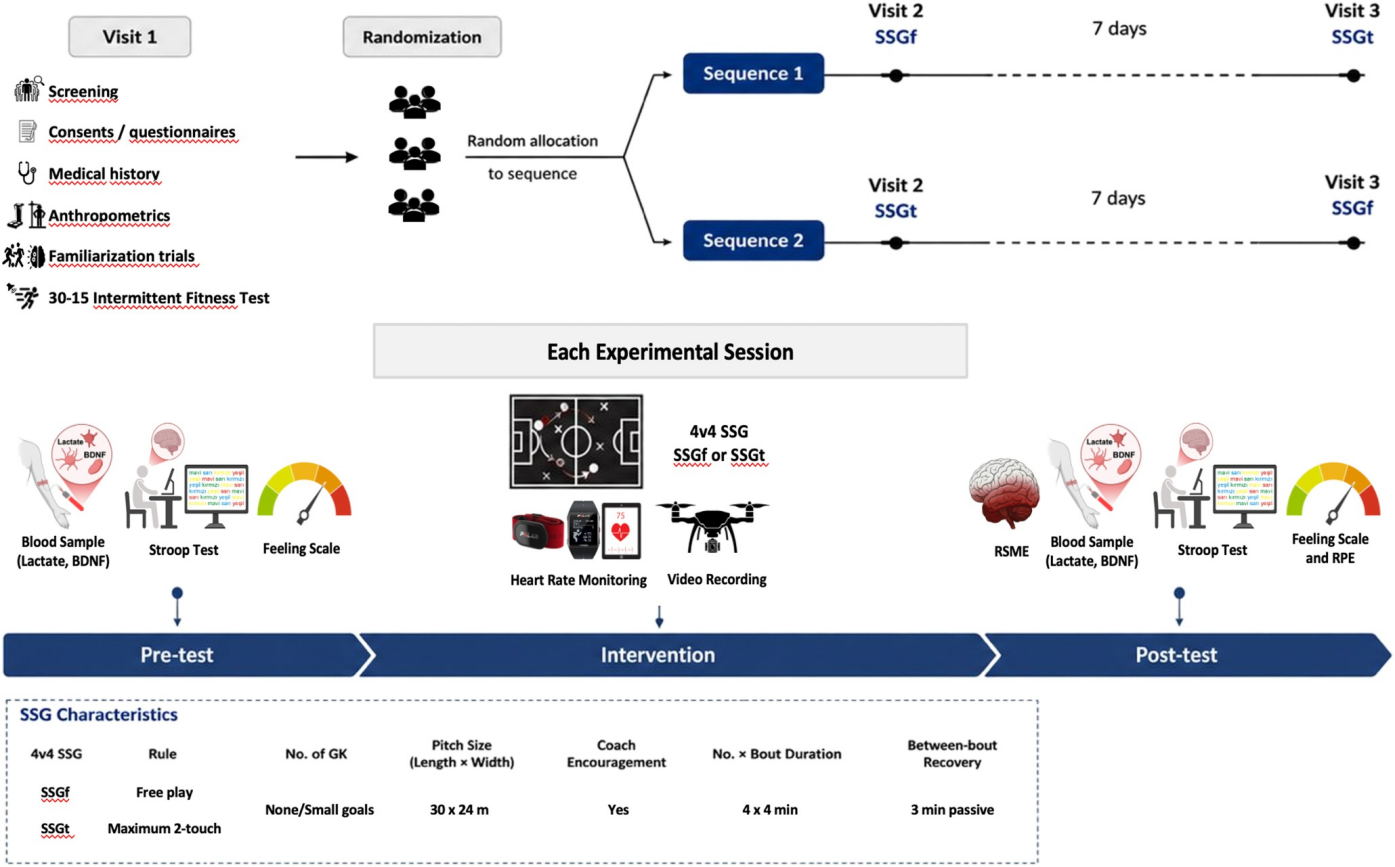
Study design. BDNF, Brain‐derived neurotrophic factor; GK, Goalkeeper; RPE, Rating of perceived exertion; RSME, Rating scale of mental effort; SSGf, Small‐sided games free‐play; SSGt, Small‐sided games with touch restriction.

A randomized crossover design was used to investigate the acute effects of SSGt and SSGf on neurochemical responses, cognitive performance, and technical actions in male soccer players. In SSGt, a maximum of two ball touches per player was imposed, whereas in SSGf, players were allowed to play without any touch restriction. The outcome measures included serum BDNF levels, blood lactate concentration, technical actions (interceptions, lost balls, successful and unsuccessful passes, and successful and unsuccessful shots), and executive function. The study consisted of a familiarization session followed by two experimental SSG sessions, as shown in Figure 1. During the familiarization session, participants were introduced to the Stroop test, testing procedures, and experimental protocol to minimize potential learning effects. Given their competitive level and training background, all players were already accustomed to SSG formats involving touch restrictions. Each participant completed both formats using a randomized counterbalanced crossover design. A 7‐day washout period was implemented between sessions to ensure sufficient physiological recovery and minimize potential carryover effects.

### 2.2 Participants

Thirty‐two male soccer players (age: 21.7 ± 1.7 years, body height: 178 ± 4 cm, body mass: 76.3 ± 3.6 kg; body mass index: 24 ± 1 kg·m^−2^; body fat percentage: 12 ± 5.95; VO_2_max: 50.7 ± 3.7 mL · kg^−1^ · min^−1^) voluntarily participated in this study during the 2024–2025 off‐season period (Table 1). As part of their off‐season program, they completed prescribed individual training sessions 5 days per week. All participants were contracted professional players recruited from the same soccer club. The sample comprised all available outfield players who were regularly involved in the club's off‐season training program and met the inclusion criteria. Recruiting players from a single training squad was intended to reduce between‐team variability in training background, tactical familiarity, coaching exposure, and habitual SSG experience. The players had at least 10 years of soccer training experience and were recruited from the same team competing in the Turkish Professional Third Division. Participants were included if they met the following criteria: medical clearance for participation, no recent use of medications or supplements known to enhance aerobic or anaerobic performance, a Mini‐Mental State Examination (MMSE) score ≥ 26, and a Beck Depression Inventory (BDI) score of ≤ 9. The exclusion criteria comprised smoking, alcohol or drug use, use of psychoactive medication, a history of depression or neurological disease, any lower extremity injury within the previous 6 months resulting in ≥ 28 days of time loss or ongoing functional limitations, and the presence of neuromotor or musculoskeletal disorders.

**TABLE 1:** Participant characteristics.

Variables	Mean and SD
Age (year)	21.7 ± 1.7
Height (cm)	178 ± 4.0
Body mass (kg)	76.3 ± 3.6
BMI (kg/m^2^)	24.0 ± 1.0
VO_2_max (mL·kg^−1^ · min^−1^)	50.7 ± 3.7
Body fat percentage (%)	12.0 ± 6.0

*Note:* Values are presented as mean ± standard deviation (SD).

Abbreviations: BMI, Body mass index; VO_2_max, Maximum oxygen uptake.

The sample size was estimated using G*Power (version 3.1.9.7; University of Düsseldorf, Düsseldorf, Germany) for a repeated‐measures ANOVA (within–between interaction). Based on previously reported BDNF responses to acute exercise (effect size *f* = 0.30) reported by Devenney et al. (2019). The analysis was conducted with *α* = 0.05, power = 0.80, 2 conditions, and 2 measurements (*ε* = 1). The analysis indicated a required total sample size of 24 participants (Fcrit = 4.30, df1 = 1, and df2 = 22). However, to account for potential dropouts and increase statistical robustness, the study was conducted with 32 participants in total.

Before signing the informed consent form, the participants were informed of the research procedures, benefits, and risks. Ethical approval was obtained from the Tokat Gaziosmanpaşa University Research Ethics Committee (24.12.2024/21‐03), and the trial was retrospectively registered at ClinicalTrials. gov (NCT07395765), in accordance with the Declaration of Helsinki.

**FIGURE 2:**
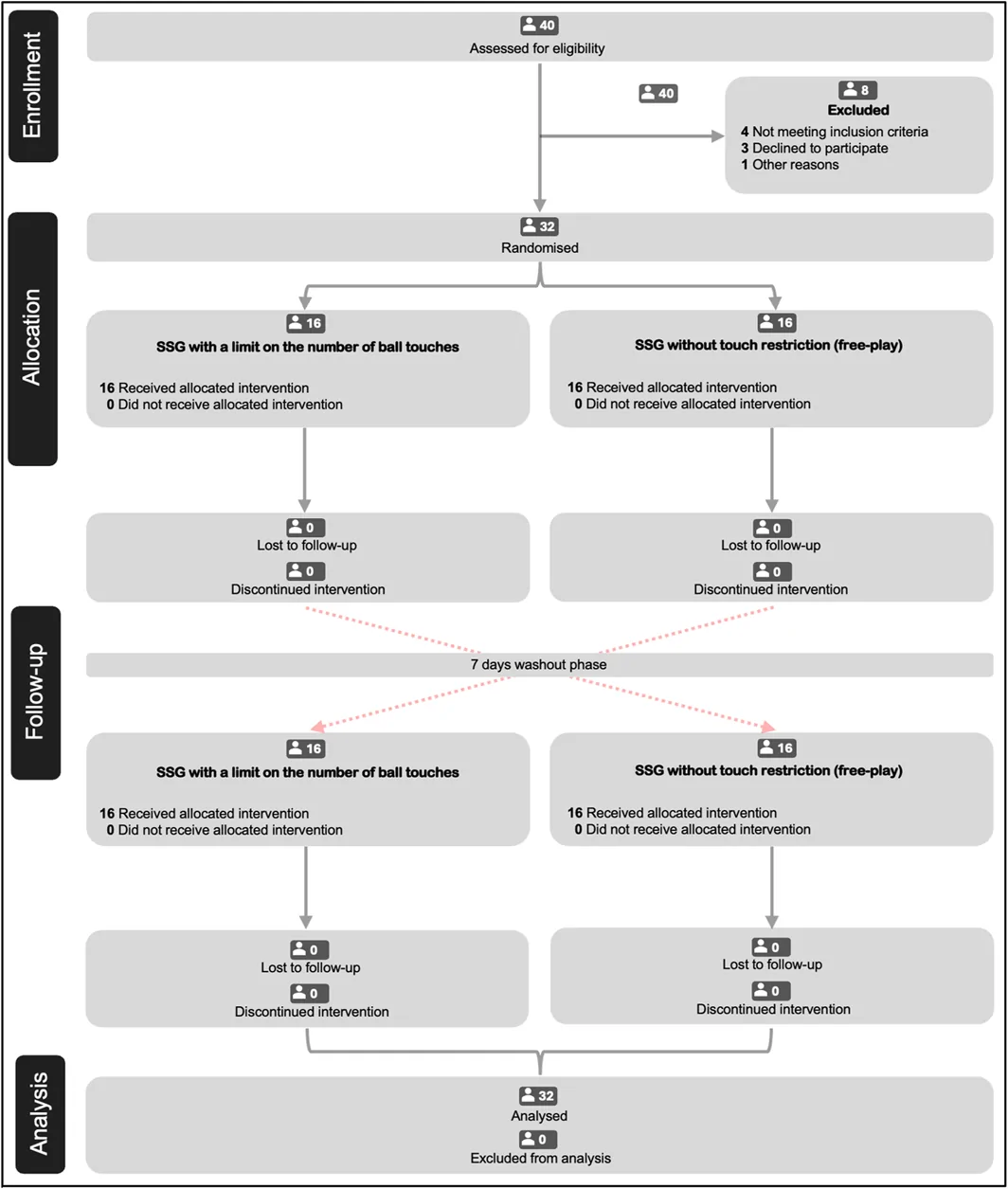
The consolidated standards of reporting trials (CONSORT) flowchart. SSG, Small‐sided games.

Participants were provided verbal information on the potential risks and benefits of the study during the initial briefing. Written informed consent was obtained before participation, and participants' willingness to continue was confirmed prior to each experimental session. The study design and reporting were conducted in accordance with the CONSORT 2025 statement (Hopewell et al. 2025) and relevant CONSORT extensions for randomized crossover trials (Dwan et al. 2019) and non‐pharmacological interventions (Boutron et al. 2017) (Figure 2). Participants were randomly assigned (1:1) to one of two counterbalanced condition orders (SSGt→SSGf or SSGf→SSGt) using a computer‐generated random sequence (www.randomizer.org). The allocated condition order was concealed until the completion of the baseline procedures. Given the within‐subject and non‐pharmacological nature of the SSG formats, blinding the participants and on‐field staff was not feasible. The participants were not informed of the specific study hypotheses. Laboratory analyses (serum BDNF and blood lactate) were performed using de‐identified data. Blinding of technical‐action coders was not feasible due to the obvious rule differences between formats; however, technical actions were coded offline from video recordings using predefined operational definitions and standardized coding criteria.

### 2.3 Procedures

During the first visit, body mass and body fat percentage were measured using bioelectrical impedance analysis (BC‐418, Tanita, Tokyo, Japan). Following the anthropometric assessments, resting heart rate (HRrest) was recorded for each participant. Subsequently, to mitigate potential learning effects, all participants underwent a familiarization session with Stroop Test and SSG formats. Finally, the participants completed a reliable assessment of team‐sport‐specific cardiorespiratory fitness using the 30–15 Intermittent Fitness Test (30–15 IFT), in accordance with the standardized procedure described by Buchheit (2008). The running velocity of the last fully completed stage was recorded as the maximum running speed (VIFT). Maximum oxygen consumption (VO_2_max) was then estimated using VIFT, age (A), gender (G, 2 for male), and body mass (BM), according to the equation proposed by Buchheit (2008):

$$VO^{2}\;\max\left(mL\cdot kg^{-1}\cdot min^{-1}\right)=28.3-\left(2.15\times G\right)-\left(0.71\times A\right)-\left(0.0357\times BM\right)+\left(0.0586\times A\times VIFT\right)+\left(1.03\times VIFT\right)$$

During the two subsequent visits, each participant completed both SSGt and SSGf sessions, with one session (either SSGt or SSGf) completed during visit two and the other during visit three. The sequence of sessions was randomized and counterbalanced so that half of the participants performed the touch‐restricted condition first, whereas the remaining participants began with the free‐play condition. Each session started with a standardized 15‐min warm‐up, including light running, dynamic stretching, ball‐based technical drills, and acceleration, deceleration, and agility exercises (Jäger et al. 2025). This was followed by four 4‐min bouts of SSG interspersed with 3‐min passive recovery periods. The total duration of each session was 40 minutes, comprising a 15‐min warm‐up, 16 minutes of active play, and 9 minutes of passive recovery. Venous blood samples were obtained, and cognitive performance (Stroop task and mental effort) was assessed immediately before and after each session. Heart rate was continuously monitored, and all sessions were video recorded for subsequent technical analysis. In addition, psychophysiological responses were assessed after each session using ratings of perceived exertion (RPE) on the Borg 6–20 scale (Arney et al. 2019) and affective valence on the Feeling Scale (FS) (Figure 1).

All SSG sessions were performed between 10:00 and 12:00 to control for potential circadian influences and were conducted on a natural grass surface during the off‐season period. Sessions were supervised by certified strength and conditioning coaches holding UEFA licenses to ensure protocol consistency. Throughout the study, players continued their usual off‐season training routine. Participants were instructed to refrain from strenuous physical activity for 48 hours prior to testing, to avoid alcohol and caffeine for at least 24 hours, and to obtain a minimum of 7–8 hours of sleep before each session. They were also asked to maintain their habitual diet and daily lifestyle throughout the study period. Environmental conditions were consistent across sessions, with temperatures ranging from 18°C to 21°C and relative humidity between 37% and 40%.

### 2.4 Small‐Sided Games

Both experimental conditions used a 4v4 SSG format to provide a standardized game‐based training task across conditions (Ueda et al. 2023). All SSG were performed on a 30 × 24 m pitch (720 m^2^), corresponding to a relative area of 90 m^2^ per player. Following a standardized 15‐min warm‐up, participants completed four 4‐min bouts separated by 3‐min passive recovery intervals (Figure 1). To maintain continuous play and minimize interruptions, multiple FIFA‐approved size 5 balls, with a standard mass of 410–450 g, were positioned around the pitch, and two coaches ensured rapid ball replacement when necessary. The games were played without goalkeepers, using small goals measuring 1.2 × 0.8 m and positioned centrally on each end line. Players were instructed to perform at maximal effort throughout the sessions and received standardized verbal encouragement without technical or tactical feedback (Birinci, Pancar, and Soylu 2025). To promote competitiveness, participants were encouraged to score as many goals as possible in the opposing small goal. No additional scoring rules, such as a minimum number of passes or zone‐entry requirements, were applied. Players' primary playing positions were recorded and considered during team allocation. To minimize potential imbalance between teams, SSG teams were formed by the coaching staff based on players' usual playing positions and the coach's assessment of their technical, tactical, and physical abilities, in line with previous SSG research using coach‐based criteria to constitute balanced teams (Torrents et al. 2016).

The free‐play condition (SSGf) was performed without task constraints. In contrast, the touch restriction condition (SSGt) required players to release the ball within a maximum of two touches (e.g., pass or shot). Any violation of this rule (> 2 touches) resulted in immediate turnover of possession to the opposing team to maintain game flow. Standard football rules were applied, except that goalkeepers, the offside rule, and corner kicks were not used. When the ball left the playing area, coaches restarted play immediately using replacement balls positioned around the pitch. After goals, play was restarted from the scoring team's own goal line without allowing time for reorganization. Two assistant coaches acted as timekeepers and referees, ensuring adherence to the respective conditions during each bout.

### 2.5 Cognitive Performance

Cognitive performance was evaluated using the Stroop Color and Word Test—TBAG Form, standardized for the Turkish adult population (Karakaş et al. 1999). The test assesses key executive functions, particularly inhibitory control under conditions of cognitive interference, alongside selective attention, processing speed, and cognitive flexibility. The protocol comprised five standardized conditions presented on a computer screen, requiring verbal responses: (1) reading color words in black ink, (2) reading color words in colored ink, (3) naming the color of patches, (4) naming the ink color of neutral stimuli (e.g., “XXXX”), and (5) naming the ink color of incongruent words. Responses were recorded in real time by the examiner using the TBAG scoring sheet, including errors and self‐corrections. Participants were instructed to respond as quickly and accurately as possible and to correct errors immediately. Completion time (s), including correction time, was recorded for each condition, along with error and self‐correction counts. All five Stroop conditions were analyzed. In line with the TBAG Form scoring rationale, completion time in Condition 5 was treated as the main color‐word interference outcome because this section represents the critical interference condition of the test, whereas the other conditions serve as control conditions for reading speed and color naming (Karakaş et al. 1999). Testing was standardized in a quiet well‐lit environment, and a brief familiarization trial was provided before each session. Mental effort was quantified using the Rating Scale of Mental Effort (RSME), a 0–150 mm vertical visual analogue scale frequently used in sport psychology research (Fuster et al. 2021). Participants rated their perceived mental effort immediately after each SSG.

### 2.6 Technical Performance

Technical actions were recorded in 4K resolution using a drone‐mounted camera (DJI Mini 5 Pro, Shenzhen, China) operated by an experienced match analyst. The drone was positioned above the pitch to provide a stable overhead view that captured the entire playing area and all players throughout each bout. Technical performance was analyzed using E‐Analyze Digital Soccer Match Analysis Software (Espor Digital, Ankara, Türkiye). Technical actions were coded according to previously published procedures for technical performance analysis in small‐sided games (Owen et al. 2004; F. M. Clemente et al. 2019). Specifically, the following variables were recorded: successful passes, unsuccessful passes, interceptions, successful shots, unsuccessful shots, and lost balls. A successful pass was defined as a pass that reached and was controlled by a teammate without opponent intervention, whereas an unsuccessful pass failed to reach the intended teammate or resulted in loss of possession. An interception was recorded when a defender deliberately interrupted an opponent's pass. A successful shot was defined as an attempt resulting in a goal, whereas an unsuccessful shot included attempts that missed the target or were blocked by an opponent. A lost ball was recorded when possession was lost because of poor ball control, dispossession, or a technical error. During the 3‐min passive recovery periods between bouts, players were allowed to consume water ad libitum. To determine intra‐rater reliability, a randomly selected subset of the SSG bouts was re‐analyzed by the same analyst after a 14‐day interval. Reliability was evaluated using the intraclass correlation coefficient (ICC; two‐way random‐effects model, absolute agreement, single measures), with ICC values ranging from 0.744 to 0.987, indicating good‐to‐excellent reliability.

### 2.7 Internal/Perceptual Load

To objectively quantify internal load, heart rate (HR) was recorded continuously at 5‐s intervals throughout each session using Polar V800 units (Polar Electro Oy, Kempele, Finland) paired with Polar H10 (Polar Electro Oy, Kempele, Finland) chest straps. Mean HR values were subsequently computed for each repetition period during data processing. In parallel, blood lactate concentration and RPE were also assessed. Affective state was evaluated using the FS (Hardy and Rejeski 1989), where participants reported their immediate emotional status on a continuum ranging from −5 (very bad) to +5 (very good).

### 2.8 Biochemical Analysis

Venous blood (4 mL) was collected from the antecubital vein with participants seated, immediately prior to exercise and at each designated post‐exercise time point. Following collection, samples were transported to the laboratory under controlled conditions and processed on the same day. Samples were allowed to clot for 30 min at room temperature, then centrifuged at 3000 × g for 12 min. Serum was aliquoted and stored at −80°C until analysis. Biochemical analyses (BDNF and lactate) were subsequently conducted between 16:00 and 17:30 h using standard laboratory procedures. Serum BDNF was quantified using a commercially available sandwich ELISA kit (Cloud Clone, USCNK, Wuhan, China) in accordance with the manufacturer's instructions. Blood lactate was determined using a Super GL2 analyzer (Müller Gerätebau GmbH, Freital, Germany) based on an enzymatic–amperometric electrochemical method. The analyzer was calibrated before each measurement session according to the manufacturer's guidelines. All specimens were de‐identified during analysis, and sample codes were disclosed only after completion of the measurements. Biochemical analyses were performed by an experienced laboratory technician who was blinded to the experimental conditions.

### 2.9 Statistical Analysis

IBM SPSS Statistics (version 29.0, SPSS Inc., USA) was used for all analyses. Normality was evaluated using skewness and kurtosis values, with values between −1 and +1 considered acceptable for normal distribution. A two‐way repeated‐measures ANOVA (time × condition) was conducted to analyze BDNF, Stroop test performance, lactate, RPE, and FS scores. A one‐way repeated‐measures ANOVA (condition) was used to examine technical actions, HRmean, %HR, and mental effort. Bonferroni‐corrected tests were applied for post‐hoc comparisons. Partial eta squared ($\eta_{p}^{2}$) values were reported as effect sizes (small: 0.01 ≤ $\eta_{p}^{2}$ < 0.06; medium: 0.06 ≤ $\eta_{p}^{2}$ < 0.14; large: $\eta_{p}^{2}$ ≥ 0.14). Statistical significance was set at *p* < 0.05. Graphical visualization was performed using GraphPad Prism software.

## 3 Results

### 3.1 Serum BDNF, Mental Effort, and Cognitive Performance

Descriptive statistics for serum BDNF, mental effort, and cognitive performance are presented in Table 2, whereas their acute responses across conditions, including paired individual data points, are illustrated in Supporting Information S1: Figure S1.

**TABLE 2:** Descriptive statistics and mean differences across free‐play and touch‐restriction small‐sided game conditions.

A‐ Within‐condition responses (Pre–Post)											
Variable	SSGf				SSGt				Condition × time		
	Pre	Post	Mean diff	95% CI	Pre	Post	Mean diff	95% CI	*F*	*p*	*ηp* ^2^
Lactate (mmol/L)	1.64 ± 0.23	8.08 ± 0.37	−6.44^a^	[−6.57, −6.31]	1.59 ± 0.24	8.19 ± 0.26	−6.60^a^	[−6.72, −6.48]	2.582	0.118	0.077
Serum BDNF (ng/mL)	368.48 ± 184.03	391.30 ± 135.17	−22.82	[−109.49, 63.86]	396.44 ± 171.85	567.28 ± 265.92	−170.85^a^	[−259.40, −82.29]	7.59	0.010	0.197
Feeling scale (−5,+5)	2.63 ± 1.84	2.78 ± 2.07	−0.16	[−1.05, 0.74]	2.19 ± 2.15	1.75 ± 2.40	0.44	[−0.62, 1.50]	0.841	0.366	0.026
Stroop 1 time (s)	12.90 ± 0.51	12.86 ± 0.64	0.05	[−0.09, 0.18]	12.78 ± 0.42	12.63 ± 0.50	0.16	[−0.11, 0.41]	1.184	0.285	0.037
Stroop 2 time (s)	16.51 ± 0.46	16.48 ± 0.51	0.03	[−0.08, 0.14]	16.44 ± 0.38	16.37 ± 0.45	0.08	[−0.14, 0.30]	0.274	0.604	0.009
Stroop 3 time (s)	19.94 ± 0.66	19.89 ± 0.68	0.05	[−0.12, 0.22]	19.92 ± 0.40	19.82 ± 0.42	0.10	[−0.09, 0.29]	0.291	0.593	0.009
Stroop 4 time (s)	20.01 ± 0.93	20.27 ± 1.08	−0.26	[−0.63, 0.11]	20.03 ± 0.92	19.11 ± 0.86	0.92^a^	[0.55, 1.30]	20.517	< 0.001	0.398
Stroop 5 time (s)	30.42 ± 0.44	30.52 ± 0.36	−0.10	[−0.26, 0.06]	30.23 ± 0.45	29.57 ± 0.46	0.66^a^	[0.50, 0.82]	36.051	< 0.001	0.538

B‐ Between‐condition responses							
Variable	SSGf	SSGt	Mean diff	95% CI	*F*	*p*	*ηp* ^2^
Successful pass (*n*)	29.03 ± 1.47	44.69 ± 1.87	−15.66^a^	[−16.30, −15.01]	1113.65	< 0.001	0.973
Unsuccessful pass (*n*)	3.66 ± 0.48	3.38 ± 0.49	0.28^a^	[0.07, 0.49]	6.51	0.016	0.174
Successful shot *n*)	1.53 ± 0.51	1.81 ± 0.40	−0.28^a^	[−0.53, −0.03]	7.496	0.010	0.195
Unsuccessful shot ((*n*)	1.66 ± 0.48	1.53 ± 0.51	0.13	[−0.07, 0.32]	0.000	1.000	0.000
Interception (*n*)	3.13 ± 0.34	1.84 ± 0.37	1.28^a^	[1.09, 1.47]	36.270	< 0.001	0.539
Lost ball (*n*)	1.72 ± 0.52	1.44 ± 0.50	0.28^a^	[0.05, 0.51]	10.62	0.003	0.255
HRmean (bpm)	168.79 ± 6.96	170.44 ± 5.66	−1.65	[−4.52, 1.22]	2.659	0.113	0.079
%HR (bpm)	84.14 ± 3.77	84.95 ± 3.03	−0.82	[−1.85, 0.22]	2.594	0.117	0.077
Mental effort (0–150 mm)	51.72 ± 7.89	85.13 ± 12.18	−33.41^a^	[−37.90, −28.91]	229.692	< 0.001	0.881
RPE (6–20 score)	16.88 ± 0.71	17.16 ± 0.72	−0.28 ± 0.16	[−0.60 ± 0.04]	3.207	0.083	0.094

*Note:* Values are presented as mean ± standard deviation (SD).

Abbreviations: BDNF, brain‐derived neurotrophic factor; bpm, beats per minute; CI, confidence interval; mm, millimetres; n, number; ng/mL, nanograms per millilitre; RPE, Rating of perceived exertion; s, seconds; SSGf, free‐play small‐sided games; SSGt, small‐sided games with touch restriction.

^a^ Indicates a statistically significant difference.

As presented in Table 2, serum BDNF levels exhibited a significant condition × time interaction, suggesting that temporal changes in BDNF differed significantly between conditions. There was no significant difference between SSGf and SSGt (MD = −27.95, 95% CI −124.30 to 68.39, *p* = 0.558) at pre‐exercise. A significant difference was observed, with SSGt showing higher values than SSGf (MD [SSGf − SSGt] = −204.12, 95% CI −289.84 to −118.41, *p* < 0.001) at post‐exercise (Supporting Information S1: Figure S1A). A highly significant main effect of condition (SSGf vs. SSGt) on mental effort level was observed, indicating greater mental effort in SSGt (Supporting Information S1: Figure S1B). Stroop 4 Completion time (sec) demonstrated a significant condition × time interaction (Supporting Information S1: Figure S1C). Bonferroni‐adjusted pairwise comparisons showed that there was no difference between SSGf and SSGt (MD = −0.021, *p* = 0.919) at pre‐exercise. But SSGt differed significantly from SSGf (MD = 1.164, *p* < 0.0001; 95% CI [0.708, 1.620]) at post‐exercise, indicating better performance in SSGt. Stroop 5 Completion time (sec) revealed a significant condition × time interaction (Supporting Information S1: Figure S1D). Bonferroni‐adjusted comparisons indicated no significant difference between SSGf and SSGt (MD = 0.198, *p* = 0.090) at pre‐exercise. But SSGf and SSGt differed significantly (MD = 0.958, *p* < 0.0001; 95% CI [0.734, 1.182]) at post‐exercise, indicating better performance in SSGt. There were no statistically significant differences between conditions in the number of corrections and errors across all sections of the Stroop Test (1–5), both before and after exercise (*p* > 0.05). Descriptive statistics for Stroop 1, 2, and 3 Completion times (sec) are presented in Table 2. Stroop 1 Completion time (sec) showed no significant condition × time interaction. Stroop 2 Completion time (sec) showed no significant condition × time interaction. Stroop 3 Completion time (sec) revealed no significant condition × time interaction.

### 3.2 Technical Actions

Descriptive statistics for technical actions are presented in Table 2, whereas their acute responses across conditions, including paired individual data points, are illustrated in Supporting Information S1: Figure S2.

Successful pass number revealed a significant main effect of condition, indicating that SSGt resulted in a greater number of successful passes than SSGf (Supporting Information S1: Figure S2A). Lost ball number revealed a significant main effect of condition on the outcome variable; the number of lost balls was lower in SSGt than in SSGf (Supporting Information S1: Figure S2B). Unsuccessful pass number revealed a significant main effect of condition; the number of unsuccessful passes was lower in SSGt than in SSGf (Supporting Information S1: Figure S2C). Interception number demonstrated a significant main effect of condition (SSGf vs. SSGt) on the outcome variable; the number of interceptions was greater in SSGf than in SSGt (Supporting Information S1: Figure S2D). Successful shot number revealed a significant main effect of condition; the number of successful shots was significantly greater in SSGt than in SSGf (Supporting Information S1: Figure S2E). Unsuccessful shot number showed no main effect of condition (SSGf vs. SSGt) on the outcome variable (Supporting Information S1: Figure S2F).

### 3.3 Physiological and Perceptual Responses

Descriptive statistics for lactate, FS, RPE, and heart rate responses are presented in Table 2, whereas their acute responses across conditions, including paired individual data points, are illustrated in Supporting Information S1: Figure S3. Lactate levels showed a significant main effect of time (pre vs. post) (F(1,31) = 15329.595, *p* < 0.001, partial *η* ^2^ = 0.998), but the condition × time interaction was not significant (Supporting Information S1: Figure S3A). Both SSGf and SSGt showed significant pre–post changes, supporting a robust main effect of time irrespective of conditions. FS revealed no statistically significant condition × time interaction (Supporting Information S1: Figure S3B). No significant main effects of condition were observed for HR% (Supporting Information S1: Figure S3C), mean HR (Supporting Information S1: Figure S3D), or RPE (Supporting Information S1: Figure S3E).

## 4 Discussion

The present study examined the acute effects of two 4v4 SSG formats on neurochemical, cognitive, and technical responses in young male soccer players. Overall, the findings were broadly consistent with our hypotheses. Compared with SSGf, SSGt was associated with greater acute serum BDNF responses, greater perceived mental effort, and faster completion time in the incongruent color‐word interference condition of the Stroop test, whereas errors and corrections did not differ between formats. The two formats also produced different technical performance with SSGt increasing successful passes and shots and reducing unsuccessful passes and lost balls. Importantly, these differences occurred despite comparable internal‐load responses. The findings provide novel insight into the possible potential role of touch restriction as a task constraint for shaping cognitive, peripheral neurochemical, and technical‐action responses during soccer‐specific SSG.

### 4.1 Serum BDNF Levels

A growing body of evidence indicates that acute and chronic exercise is a well‐established stimulus for increasing circulating BDNF levels across a wide range of populations, including adolescents, young adults, and older individuals (Dinoff et al. 2017; Erickson et al. 2011; Szuhany et al. 2015). Nevertheless, despite the consistency of these findings in general populations, evidence remains limited in trained athletic populations, particularly in studies utilizing soccer‐specific SSG formats. Soccer is relevant in this context because it combines physical exertion with continuous cognitive demands, making it a potentially important model for studying exercise‐related neurocognitive responses.

Studies investigating exercise‐induced BDNF responses in soccer players remain limited and yield inconsistent findings. A systematic review by Gutiérrez ‐Vargas et al. (2021) concluded that current evidence is insufficient to draw definitive conclusions regarding soccer‐specific exercise effects on BDNF levels, largely due to heterogeneity in exercise protocols, sampling procedures, and participant characteristics. Among the limited available studies, Yang et al. (2012) reported a significant acute increase in BDNF following a 90‐min competitive match in adolescent males. However, full match play and SSG may differ in terms of physiological load, tactical complexity, and technical action profiles, which may lead to distinct neurobiological responses. Evidence specific to SSG is scarce and has predominantly shown no significant BDNF changes. For instance, Williams et al. (2020) reported no acute BDNF response following a soccer‐specific session incorporating technical drills and a 5‐a‐side SSG, potentially due to moderate exercise intensity (∼75% HRmax). Similarly, our previous work demonstrated that free‐play 4v4 SSG formats do not elicit significant BDNF increases (Birinciet al. 2025). In contrast, the introduction of an additional task constraint (i.e., limiting ball touches) in the same SSG format resulted in a significantly greater BDNF response. These findings suggest that exercise conditions combining physical demands with increased cognitive load may exert a synergistic effect on acute BDNF release. Notably, despite comparable internal load responses, only the cognitively constrained condition showed significant BDNF elevations, suggesting that task‐related contextual factors may contribute to acute BDNF responses beyond exercise intensity alone. Consequently, exercise‐induced changes in BDNF appear to be highly sensitive to contextual modulation. Variations in protocol design (intensity, duration, modality), participant characteristics (sex and training status), and sampling methodology collectively contribute to the divergent findings reported in the literature (Behrendt et al. 2021; Birinci et al. 2026; Dinoff et al. 2017).

The present findings should be interpreted within the broader distinction between open skill exercise (OSE) and closed‐skill exercise (CSE). OSE, characterized by externally paced, unpredictable environments requiring continuous perceptual monitoring and rapid decision‐making, has been proposed to elicit greater neurotrophic responses compared with CSE (Hung et al. 2018). This view is based on the premise that simultaneous physical and cognitive engagement may be associated with larger acute neurotrophic and better cognitive responses than physical exertion alone (Voelcker‐Rehage and Niemann 2013; Zhu et al. 2020). However, empirical evidence remains mixed (Birinci et al. 2024). For example, in a non‐soccer comparison of OSE and CSE with equivalent relative intensity, Behrendt et al. (2021) reported that both modalities acutely increased peripheral BDNF and related biomarkers, although longer‐term changes tended to favor OSE. Likewise, cross‐sectional data comparing athletes engaged in OSE versus CSE sports showed no significant difference in basal BDNF between groups (Gökçe et al. 2021), suggesting that the OSE advantage may not generalize across populations or study designs. Taken together, these findings indicate that categorizing exercise as OSE or CSE may be insufficient on its own; rather, the specific task properties embedded within an exercise (e.g., constraint‐induced spatiotemporal demands and executive‐control requirements) may determine the magnitude of neurotrophic responses. Although free‐play SSG are inherently OSE in nature, they may allow players to rely on habitual motor patterns and automatized decision‐making (Coutinho et al. 2022). By contrast, imposing a touch restriction may increase spatiotemporal pressure and require greater inhibitory control, anticipation, and rapid option selection. This manipulation likely increases cognitive engagement beyond that of unconstrained play, thereby creating a more cognitively demanding game‐based condition. This interpretation is also consistent with the broader concept of environmental enrichment, in which novelty and cognitive stimulation are considered relevant to cognitive outcomes and BDNF‐related responses (Sadegzadeh et al. 2020). The present findings suggest that, within soccer‐specific SSG, the task constraint itself may be an important factor in shaping acute BDNF responses. Manipulating SSG rules to increase cognitive load, such as touch restriction, may represent a practical strategy for coaches aiming to target not only physical and technical outcomes but also neurobiological processes related to cognitive function and brain plasticity.

### 4.2 Cognitive Responses

Beyond the neurochemical responses, the cognitively constrained SSG also resulted in greater improvements in executive performance, as reflected by faster Stroop completion time. The Stroop test primarily assesses inhibitory control and selective attention (Karakaş et al. 1999), which are relevant to game intelligence and adaptive behavior in soccer (Haugan et al. 2025). In this context, the touch‐restricted format may have increased executive demands by requiring players to process game information and select actions with fewer opportunities for additional ball manipulation and a greater need for early option selection during SSG.

To date, research examining the acute cognitive effects of soccer‐specific exercise remains limited. Evidence from SSG protocols suggests that exercise intensity and task characteristics may differentially influence executive outcomes. For instance, Lind et al. (2019) demonstrated that high‐intensity 3‐a‐side SSGs improved inhibitory control and attention‐related neurophysiological markers compared with moderate‐intensity formats in children. Longitudinal evidence also indicates that structured football programs incorporating SSG may enhance visual discrimination and selective attention (Alesi et al. 2015). Similarly, acute SSG formats have been shown to improve attentional performance in adolescents (Hammami et al. 2023) and visuomotor processing speed in young soccer players (Birinci et al. 2025). However, findings are not uniformly positive. Skala and Zemková (2023) reported faster reaction times but increased response errors following a 4‐a‐side SSG, suggesting that fatigue‐related factors may offset potential cognitive benefits. Collectively, these findings indicate that soccer‐specific cognitive responses may depend on exercise intensity, duration, and task structure, highlighting the importance of contextual variables in interpreting executive outcomes.

SSG inherently stimulate perceptual–cognitive processes due to their dynamic and interactive structure (Davids et al. 2013; Rodrigues et al. 2022). Limiting ball touches may increase attentional and action‐selection demands during SSG, which is consistent with previous evidence showing that touch restrictions modify technical behavior and decision‐making patterns (F. Clemente and Sarmento 2020; Coutinho et al. 2022). In the present study, the higher RSME scores observed in SSGt support the interpretation that the touch‐restricted format imposed greater cognitive load than free play. This interpretation is also supported by recent evidence showing that task constraints may partly dissociate cognitive demand from physical effort; for example, González‐Fernández et al. (2026) reported higher mental workload during smaller‐pitch SSG despite higher rating of perceived exertion in larger‐pitch formats. Although their manipulation involved pitch size rather than touch restriction, their findings suggest that the cognitive demands imposed by SSG constraints may not necessarily parallel internal load responses. Acute exercise has been associated with transient enhancements in executive functions, particularly inhibitory control (Piepmeier and Etnier 2015), and cognitively demanding or coordinatively complex activities may elicit more pronounced effects than repetitive closed‐skill tasks (Voelcker‐Rehage and Niemann 2013). Importantly, although internal load markers were comparable between conditions, only SSGt improved executive performance. This suggests that internal load alone, as reflected by heart rate and blood lactate responses, may not fully explain the observed Stroop improvement; rather, the combination of physical exertion and elevated cognitive demands may have provided a stronger stimulus for executive function performance.

The parallel increases in BDNF and Stroop performance observed in the SSGt may reflect converging responses to elevated cognitive engagement. BDNF has been implicated in synaptic plasticity and prefrontal‐dependent executive processes (Erickson et al. 2011), and acute exercise‐induced BDNF elevations have been proposed as a potential mechanism underlying transient improvements in cognitive performance (Dinoff et al. 2017; Szuhany et al. 2015). Although a direct causal pathway cannot be established, the present findings remain consistent with a possible link between acute neurochemical and executive‐performance responses. This pattern may provide a useful basis for further examining neurochemical and executive‐function responses to cognitively enriched soccer‐specific exercise.

### 4.3 Technical Actions

The touch‐limited SSG condition produced a distinct technical‐action profile compared with the free‐play format. Specifically, SSGt resulted in higher numbers of successful passes and successful shots, together with fewer unsuccessful passes and lost balls. Previous research has shown that limiting ball touches alters technical behavior by increasing passing involvement and reducing opportunities for prolonged ball possession as well as individual dribbling actions (F. Clemente and Sarmento 2020; Coutinho et al. 2022). In this context, the increase in successful passes and shots observed in the constrained condition may reflect a shift toward faster ball circulation and more direct attacking actions. Importantly, these technical changes occurred despite the higher perceived mental effort reported in SSGt, suggesting that the additional cognitive demands imposed by the touch restriction did not compromise technical effectiveness. These findings indicate that increasing cognitive demands within an ecologically valid game context does not necessarily compromise technical effectiveness when implemented through a soccer‐specific task constraint such as limiting ball touches.

In practical terms, the maximum two‐touch rule required players to perceive available passing or shooting options, regulate ball possession within the imposed rule, and execute technical actions with limited touches in the ongoing game context. These demands are consistent with executive processes such as inhibitory control, selective attention, and rapid response selection, which are relevant to game intelligence and adaptive behavior in soccer (Badin et al. 2016; Haugan et al. 2025). Accordingly, the higher perceived mental effort observed in SSGt supports the interpretation that the touch‐restricted format created a more cognitively demanding game‐based condition than free play. This may help explain why SSGt elicited more favorable executive function responses without compromising technical effectiveness.

The present study has limitations that should be acknowledged. BDNF concentrations were assessed at a single post exercise time point. Given that circulating BDNF levels are time‐sensitive and may peak approximately 15 min after exercise (Birinci et al. 2026), the selected sampling window may not have captured the maximal response across conditions, although an acute increase was still detectable following the touch‐restriction format. In addition, BDNF was measured only in serum, which may reflect platelet‐derived release and is affected by clotting‐related factors (Gejl et al. 2019), whereas plasma BDNF and platelet indices were not assessed. Plasma volume changes were also not estimated; therefore, potential hemoconcentration effects cannot be excluded. Regarding cognitive assessment, although the RSME provides ecologically valid insight into perceived cognitive load, subjective mental‐effort ratings do not directly quantify neural activation or cortical engagement (Otto et al. 2014). Furthermore, reliance on a single cognitive performance test (i.e., Stroop task), may have influenced the observed exercise related effects. The sample was also limited to young male soccer players; therefore, the findings may not generalize to other populations, including female players, youth athletes at different stages of neurodevelopment, or older adults. Future studies should incorporate a broader range of cognitive assessments, additional neurobiological markers, and multiple post exercise sampling time points to more comprehensively characterize the neurocognitive responses to game based training. Long term investigations examining repeated exposure to cognitively enriched SSG formats are warranted to determine whether the acute neurotrophic and executive benefits observed translate into sustained adaptations over time.

## 5 Conclusion

In conclusion, imposing a touch restriction during 4v4 SSG was associated with greater acute increases in serum BDNF and faster completion time in the incongruent color‐word interference condition of the Stroop test compared with free‐play SSG. Moreover, the cognitively constrained format was associated with greater technical effectiveness, particularly in successful passing and shooting actions. These findings suggest that task design may be important for creating cognitively stimulating soccer training environments. Accordingly, coaches may consider strategically increasing cognitive demands within SSG by limiting ball touches to support acute neurotrophic responses, executive function performance, and technical effectiveness. This approach may be particularly relevant in youth or skill‐development settings, where cognitively stimulating practice environments could contribute to technically and cognitively engaging practice conditions.

## Author Contributions

All listed authors should have contributed to the manuscript substantially and have agreed to the final submitted version.

## Funding

The authors have nothing to report.

## Ethics Statement

Before signing the informed consent form, subjects were informed of the research procedures, benefits, and risks. Ethical approval was obtained from the Tokat Gaziosmanpaşa University Research Ethics Committee (24.12.2024/21‐03), and the trial was retrospectively registered at ClinicalTrials. gov (NCT07395765; https://clinicaltrials.gov/study/NCT07395765), in accordance with the Declaration of Helsinki.

## Conflicts of Interest

The authors declare no conflicts of interest.

## Supporting information


Supporting Information S1

## Data Availability

The datasets generated and/or analyzed during the current study are not publicly available due to ethical and privacy considerations to protect participant identity but are available from the corresponding author on reasonable request.
