# Supplementary material for: Touch Restriction Versus Free‐Play Small‐Sided Games in Soccer: Acute Effects on Serum BDNF, Executive Function, and Technical Actions
Source: Eur J Sport Sci. 2026 Aug 22;26(9):e70242. doi: 10.1002/ejsc.70242 (PMC13499902; doi:10.1002/ejsc.70242)
Supplement: Supplementary file 1 [file EJSC-26-e70242-s001.docx]

**Figure S-1.** Acute responses to free-play (SSGf) and touch restriction (SSGt) small-sided games. **(A)** Serum BDNF concentrations (ng/mL) before and after SSGf and SSGt conditions. **(B)** Mental effort ratings following SSGf and SSGt conditions. **(C–D)** Stroop test performance: **(C)** Stroop 4 completion time, and **(D)** Stroop 5 completion time, assessed before and after both SSG conditions. Data are presented as violin plots. BDNF: brain-derived neurotrophic factor; SSGf: free-play small-sided games; SSGt: small-sided games with touch restriction; ns: non-significant (p > 0.05); *p ≤ 0.05. ng/mL: Nanograms per millilitre.

**Figure S-2**. Technical actions in response to free-play (SSGf) and touch restriction (SSGt) small-sided games. **(A)** Successful pass number. **(B)** Unsuccessful pass number. **(C)** Successful shot number. **(D)** Unsuccessful shot number. **(E)** Interception number. **(F)** Lost ball number. Data are presented as mean ± standard deviation. SSGf: small-sided games (free-play); SSGt: small-sided games with touch limitation; ns: non-significant (p > 0.05); *p ≤ 0.05.

**Figure S-3.** Physiological and perceptual responses to free-play (SSGf) and touch restriction (SSGt) small-sided games. **(A)** Blood lactate concentration before and after SSGf and SSGt conditions. **(B)** Feeling scale responses before and after SSGf and SSGt conditions. **(C)** Mean heart rate expressed as percentage of maximum heart rate (HR%). **(D)** Mean heart rate (bpm) during SSGf and SSGt conditions. **(E)** RPE score during SSGf and SSGt conditions. Data are presented as mean ± standard deviation. RPE: Rating of perceived exertion; Ra SSGf: small-sided games (free-play); SSGt: small-sided games with touch limitation; HR: heart rate; bpm: beats per minute; mmol/L: Millimoles per liter; ns: non-significant (p > 0.05); *p ≤ 0.05.
